# Unveiling advanced green assessment of simple and cost effective spectrophotometric determination of domperidone and pantoprazole for gastrointestinal disorders

**DOI:** 10.1038/s41598-026-35089-1

**Published:** 2026-01-26

**Authors:** Eman Darweish, Magda Mohamed El-Henawee, Mona Abd Elnasser Labib, Naglaa Abdel Sattar Kabil

**Affiliations:** 1 Pharmaceutical Chemistry Department, Faculty of Pharmacy, Egyptian Russian University, Badr City, Cairo, 11829 Egypt; 2 Pharmaceutical Analytical Chemistry, Faculty of Pharmacy, Zagazig University, Zagazig, 44519 Egypt

**Keywords:** Domperidone, Pantoprazole, Deconvoluted fourier, Dual wavelength, First-order derivative, Second-order derivative, Greenness, Chemistry, Environmental sciences

## Abstract

In the pursuit of sustainable pharmaceutical analysis, Green Analytical Chemistry emerges as a vital approach to protect both human health and the environment. This study focuses on a green analytical approach for the simultaneous evaluation of Domperidone (DP) and Pantoprazole (PP), two widely used gastrointestinal drugs with distinct mechanisms and therapeutic roles. A suite of advanced spectrophotometric techniques, including the Deconvoluted Fourier method (DF), the Dual Wavelength method (DW), and both First-Order Derivative (D^1^) and Second-Order Derivative (D^2^) methods, was developed for the simultaneous determination of DP and PP. The linearity ranges were found to be (1.0– 9.0) µg/mL for both drugs; this range applies to all the proposed methods. Accuracy and reliability were confirmed via the standard addition technique. Furthermore, the performance of the suggested approaches was statistically compared with that of previously reported HPLC methods, showing their efficiency, eco-friendliness, and practical applicability. The methods were applied to laboratory-prepared combinations with varying concentrations of the two drugs to evaluate selectivity, as well as to their commercially available dosage forms. These methods were rigorously assessed for their environmental sustainability using green analytical chemistry metrics: The Analytical Eco-Scale, the Modified Green Analytical Procedure Index (MoGAPI), the Analytical Green Star Area (AGSA), Carbon footprint (CaFRI), Blueness evaluation using BAGI tools, Click Analytical Chemistry Index (CACI) and the Multi-Color Assessment (MA) Tool.

**Supplementary Information:**

The online version contains supplementary material available at 10.1038/s41598-026-35089-1.

## Introduction

Gastric motility disorders can cause symptoms such as nausea, vomiting, abdominal bloating, and heartburn, prompting patients to seek medical attention^1^. The new combination of DP and PP has become a cornerstone in the management of upper gastrointestinal disorders, offering a synergistic approach that enhances therapeutic outcomes. The importance of this combination lies not only in symptom control but also in reducing the risk of complications such as esophageal ulcers, Barrett’s esophagus, and gastric malignancies. Untreated or poorly managed upper gastrointestinal disorders pose serious health hazards, including chronic inflammation, bleeding, and a marked decline in patients’ quality of life. Thus, the DP with PP combination represents a pivotal advancement in gastroenterological therapy, contributing to improved patient outcomes and reduced disease burden.

**Fig. 1:**
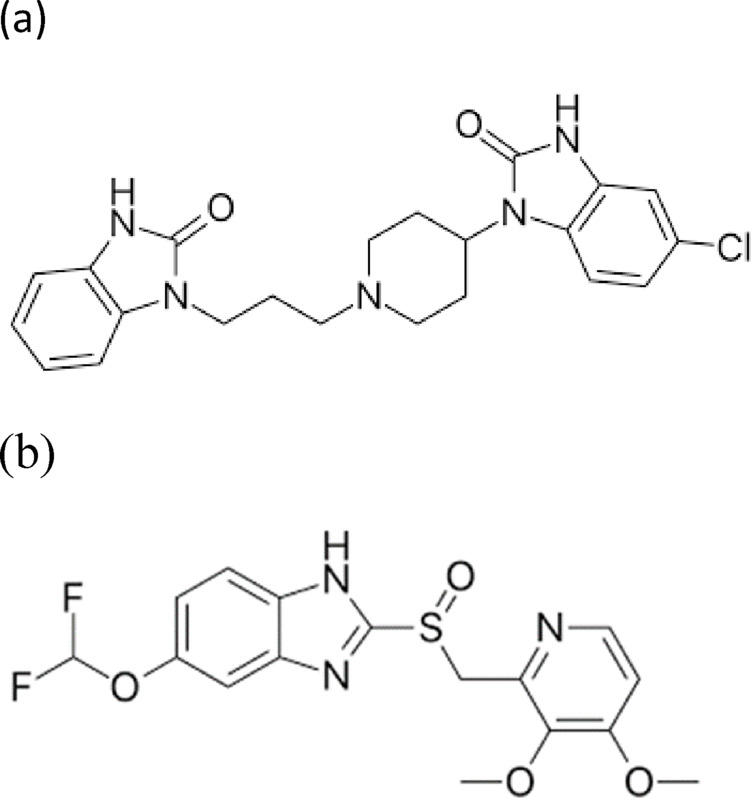
Chemical structure of (**a**) Domperidone and (**b**) pantoprazole.

Domperidone (DP; Fig. 1a) is 5-chloro-1-[1-[3-(2,3-dihydro-2-oxo-1 H-benzimidazole-1-yl)-propyl]-4-piperidinyl]-1-3-dihydro-2 H-benzimidazole-2-one. It is a peripheral dopamine D₂ receptor antagonist at the therapeutic dose of 10 mg, which is frequently taken as an antiemetic drug to treat vomiting and nausea of various causes in the short term^2^.

Pantoprazole (PP; Fig. 1b) is 5-[difluoromethoxy]-2-{[(3,4-dimethoxy-2-pyridinyl) methyl]sulfinyl}-lH-benzimidazole^2^. PP, an irreversible proton pump inhibitor, efficiently lowers stomach acid output by carrying out the last stage of the acid secretion process at a therapeutic dose of 40 mg^3^. In addition, it is utilized as an antiulcer medication. It is the first-line treatment for many individuals with acid-peptic illnesses, such as gastroesophageal reflux disease (GERD), non-erosive reflux disease (NERD), and duodenal gastric ulcers^4^.

Spectrophotometry is a readily available and affordable analytical technique, commonly used in developing country laboratories to address diverse analytical needs^5,6^. A literature review revealed a scarcity of analytical methods for determining DP and PP; chromatographic techniques^7–11^, spectrophotometric techniques such as simultaneous estimation^12^, ratio difference, ratio derivative, and mean centering^13^, Ion Mobility Spectrometry^14^, A rapid Fourier transform infrared spectroscopic method^15^, Molecular and Biomolecular Spectroscopy^16^, a three-wavelength spectrophotometric method^17^ and Electrochemical approaches^18,19^. This innovative UV spectrophotometry method offers a green, affordable, and precise method for measuring DP and PP. A comprehensive assessment further verifies its environmental benefits. This paper investigates a range of spectrophotometric techniques, including Dual-Wavelength (DW), Deconvoluted Fourier method (DF), First-order derivative method (D^1^), and Second-order derivative method (D^2^) for the precise determination of DP and PP. This work offers a time- and cost-effective solution to resolve severely overlapped spectra, validated in compliance with ICH standards^20^. The methods were validated using the standard addition technique, demonstrating their suitability for routine quality control analysis of pharmaceutical and pure substances, in terms of lower LOD and LOQ values, wider applicability to pharmaceutical formulations, and overall benefits such as simplicity, cost-effectiveness, rapid analysis, minimal solvent consumption, and enhanced greenness. These points emphasize the superiority and practical relevance of the proposed methods. No complex procedures or specialized equipment are required. The techniques demonstrated their sensitivity, yielding LOD values of 0.148 µg/mL for DM and 0.131 µg/mL for PP in the DF method, 0.193 µg/mL for DM and 0.177 µg/mL for PP in the DW method, and 0.195 µg/mL for DM, 0.153 µg/mL for PP in the D^1^ method and 0.129 µg/mL for DM, 0.102 µg/mL for PP in the D^2^ method. Greenness assessment is crucial for understanding and mitigating the environmental impact of products, processes, and policies. It helps identify and prioritize actions that minimize resource consumption, reduce pollution, and promote sustainability—the analytical eco-scale system (which is based on penalty points). The Modified Green Analytical Procedure Index (MoGAPI) provides a visual assessment of environmental impact at every stage, utilizing a pentagram-shaped pictogram with colored fields (green, yellow, and red). The Analytical Green Star Area (AGSA) employs a circular pictogram segmented into 12 sections, each color-coded from red to green. Besides utilizing Blueness assessment with BAGI tools, it presents the idea of “Blueness,” a metric that merges Greenness (derived from AGREE), method effectiveness, and analytical flexibility and efficiency. Furthermore, the “BAGI” metric, which was developed recently, was used to evaluate how well the proposed methods work in practice. Besides Carbon footprint (CaFRI), following the principle that only quantifiable data can be managed, the measurement of global carbon footprints for different products, entities, and activities reveals their greenhouse gas intensity. Click Analytical Chemistry Index (CACI) uses a color-coded pictogram to illustrate the method’s performance in various areas. A colored pictogram represents excellent performance, gray denotes middling performance, and black signifies inadequate performance or failure to meet the intended standards. In the Multi-Color Assessment (MA) Tool is the first fully integrated digital platform that is freely available and combines aspects of greenness, practicality, performance, and innovation into a real-time sustainability scoring system for analytical methods.

## Experimental

### Materials and reagents

Pure standard DP was provided by Sedico Company, located in 6th of October, Giza, and PP was provided by Biomed Pharmaceutical Company, located in Cairo, Egypt. Their purities were certified to be 99.79% and 99.76%, respectively, according to the company’s certification. Ethanol was obtained from TopChem Pharmaceutical Company, Ireland.

Pantosec-D tablet, containing DP-10 mg and PP-40 mg, was purchased from CIPLA LTD Pharmaceuticals (Mumbai, 400013 India).

### Instruments and software

A double-beam UV-vis spectrophotometer (Jasco, Japan) equipped with two identical quartz cuvettes (1 cm path length) and Spectra Manager II software was used to perform spectrophotometric observations. The scan speed was 1000 nm/min, and the spectral slit was 2 nm wide. Ultrasonic device type: Ultrasonic bath EMAG ultrasonic cleaner Emmi-20 HC, Germany.

### Standard solutions

After accurately weighing and transferring 10.0 mg of each pure standard to a 10 mL volumetric flask, two standard stock solutions (1.0 mg/mL DP and PP) were created independently. After adding ethanol and sonicating it for ten minutes, the same solvent was used to fill the volume to the desired level. By further diluting with ethanol, two working standard solutions (100.0 µg/mL DP and PP) were produced. Freshly made stock and operating standard solutions were kept in a refrigerator between 2 and 8 degrees Celsius.

### Laboratory-prepared mixture

Different laboratory-made combinations with varying ratios of DP and PP were created and tested with ethanol as a blank. The study analyzed laboratory-prepared mixes of DP and PP at various ratios and concentrations within the linear range.

### Construction of calibration curves

For both medications, standard serial solutions of DP and PP were made in ethanol at concentrations between 1.0 and 9.0 µg/mL. Following a 200–400 nm scan of the generated solutions, ethanol was used as a blank to record the zero-order absorption spectra. The spectra of this drug were saved to use in the developed method. For each method, we measured drug concentration against its corresponding absorbance.

#### Spectrophotometric methods

- Deconvoluted Fourier method for determination of DP and PP:

To deconvolute the previously saved spectra of each standard, the built-in spectrophotometer software’s Fourier deconvolution function was used, using the full width at half maximum value. The computational technique separates and reduces the bandwidth to resolve substantially overlapped spectral signals from DP and PP^21^. The approach identifies peaks by differentiating their positions in each spectrum. The end consequence was the sacrifice of the y-axis data. Light up the x-axis data to resolve severely overlapped spectra^22,23^. The DP amplitude was measured at 250.0 nm, at which PP was zero, while the PP amplitude was measured at 240.0 nm, where DP also had zero crossovers. The amplitudes of deconvoluted DP and PP spectra at particular wavelengths were plotted against their concentrations to create calibration curves. The regression equations were then calculated.

(b) Dual Wavelength method for determination of DP and PP:

The Dual Wavelength technique is based on the idea that “the absorbance difference between two spots on the mixture spectrum is directly proportional to the concentration of the identified component.“. For every drug, two wavelengths are chosen so that the absorbance difference for the second drug is zero^24^. The absorbance difference values for DP (265.0 and 301.0 nm) and PP (274.0 and 297.0 nm) were measured, where the absorbance difference for the unmeasured drug was zero.

(c) First-order derivative method for determination of DP and PP:

The derivative spectrophotometric approach is a spectrophotometric methodology that provides a valuable means for obtaining both quantitative and qualitative data from overlapping band spectra^25^.

Its base is the extraction of the wavelength-dependent first-order derivatives of absorbance from the zero-order ones. DP was measured at 290.0 nm, at which PP was zero crossing. PP was measured at 302.0 nm, at which DP was zero crossing.

(d) Second-order derivative method for determination of DP and PP:

This methodology has advantages over traditional absorbency methods, including detection of acute spectral characteristics over wide bands and improved resolution of overlapping spectra and recovery percentage^26^. Derivative spectroscopy typically yields more accurate fingerprints than standard absorbency spectra^27^. DP and PP’s second derivative spectra have zero-crossing wavelengths 304.0 nm and 287.0 nm, respectively, which can be employed for sensitive simultaneous determination.

#### Pharmaceutical dosage form assay

Ten Pantosec-D tablets^®^ were weighed, the average weight was calculated then crushed into a fine powder. Accurately weighed powder tablets equivalent to 10.0 mg of DP and 40.0 mg of PP were dissolved in 50.0 mL of ethanol sonicated for 25 min. The volume was completed with ethanol to the mark. The mixture was filtered using a 0.45 μm syringe filter. Additional dilution was done to achieve the required concentration of 1.0 µg/mL of DP and 4.0 µg/mL of PP. The determination using the developed methods was applied as indicated for each approach.

## Result and discussion

Spectrophotometry offers several advantages for analyzers, including ease of operation, low cost, availability, and environmental friendliness. Fortunately, recent years have demonstrated a spectacular improvement in the invention and implementation of smart spectrophotometric solutions^28^. Analysts face a challenging problem in quantitatively resolving mixtures with sever overlapped spectra^29^. The presented determinations here were successfully estimated for pharmaceuticals using univariate spectrophotometric methods, without the need for previous separation. The methods that were described made it possible to estimate the concentration of the chosen medication in its dose form without any overlap.

**Fig. 2:**
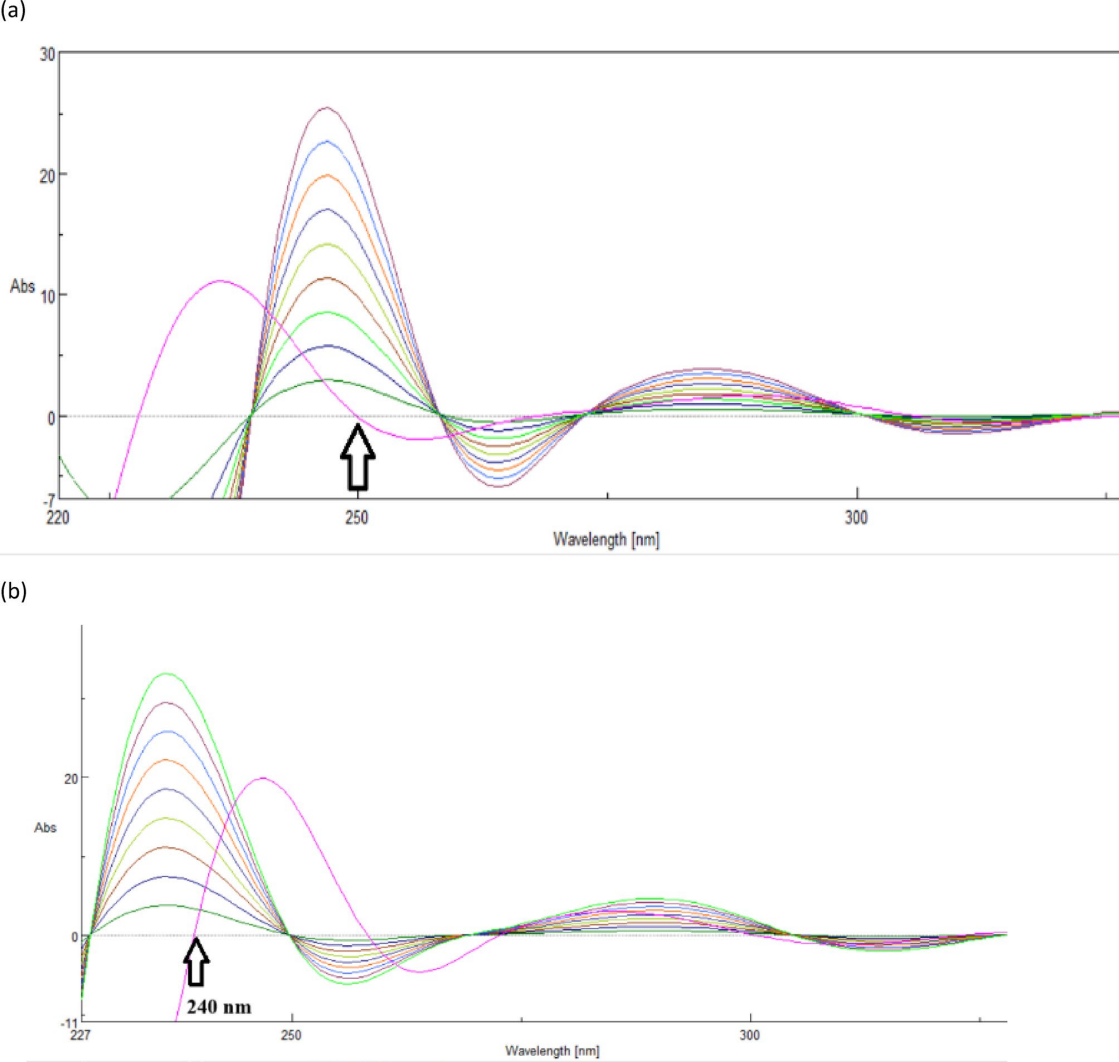
(**a**) Deconvoluted spectra of (**a**) DP (1.0–9.0 µg/mL) computed at (250.0 nm) where the zero-crossing point of the PP deconvoluted spectrum (dotted). (**b**) PP (1.0–9.0 µg/mL) determined at (240.0 nm), where no interference from DP deconvoluted spectrum (dotted).

- Deconvoluted Fourier method (DF) for determination of DP and PP:

A reliable and reasonably priced spectrophotometric technique for detecting highly overlapped spectra without previous separation is the DF method^30^. By reducing bandwidth and separating them, this computational method resolves highly overlapped spectral signals from DP and PP. This method requires little mathematical treatment of zero-order spectra. DP and PP can be examined together, and vice versa. To deconvolute, use the spectrophotometer software’s Fourier self-deconvolution tool with a Full Width at Half Maximum (FWHM) of 18. Using the Fourier approach, the DP amplitude was measured at 250.0 nm, with zero crossovers from PP. The amplitude of PP was observed at 240.0 nm with zero crossing from DP, as shown in (Fig. 2). Calibration curves were created by graphing the deconvoluted spectra amplitudes of DP and PP at 250.0 and 240.0 nm against their respective concentration values, respectively, and then the regression equations were computed and shown in (Table 1).

**Table 1:** Assay validation parameters of the developed univariate spectrophotometric methods for the determination of domperidone and pantoprazole.

Method parameters	D^0^		DF		DW		D^1^		D^2^	
	DP	PP	DP	PP	DP	PP	DP	PP	DP	PP
Linearity range (µg/mL)	1.0–9.0									
R2	0.999	0.9994	0.9999	0.9999	0.9992	0.9994	0.9992	0.9995	0.9998	0.9998
Regression equation parameters										
Slope	0.11	0.13	2.414	3.129	0.018	0.019	0.0062	0.009	0.00057	0.00076
Intercept	0.02	0.04	-0.0004	0.002	-0.0011	0.0038	0.0005	0.0006	-5.833	0.00023
Correlation coefficient (r)	0.9994	0.9996	0.9999	0.9999	0.9996	0.9997	0.9996	0.9998	0.9997	0.9998
Accuracy (Mean ± SD)	100.63 ± 0.44	99.49 ± 0.69	100.49 ± 0.42	100.06± 0.38	100.49 ± 0.58	100.36 ± 0.70	100.36 ± 0.44	99.95 ± 0.79	100.19 ± 0.55	100.42 ± 0.72
Precision										
(± %RSD)a	0.26	0.76	0.28	0.07	0.28	1.16	0.54	0.38	0.29	0.45
(± %RSD)b	0.44	1.90	0.41	0.35	0.78	0.89	1.01	0.72	0.16	0.77
LOD (µg/mL)	0.237	0.178	0.148	0.131	0.193	0.177	0.195	0.153	0.129	0.102
LOQ (µg/mL)	0.718	0.539	0.448	0.397	0.585	0.535	0.590	0.464	0.392	0.309

Repeatability (RSD a) (*n* = 3). %RSD using three concentrations within the linear range for each standard, repeated three times within the same day.

Intermediate (RSD b) (*n* = 3). %RSD using three concentrations within the linear range for each standard repeated three times within three successive days.

DF of DP = 2.4135 C + 0.00047 *r* = 0.9999

DF of PP = 3.129 C + 0.0021 *r* = 0.9999

where r is the correlation coefficient and C is the drug’s concentration (µg/ml).

**Fig. 3:**
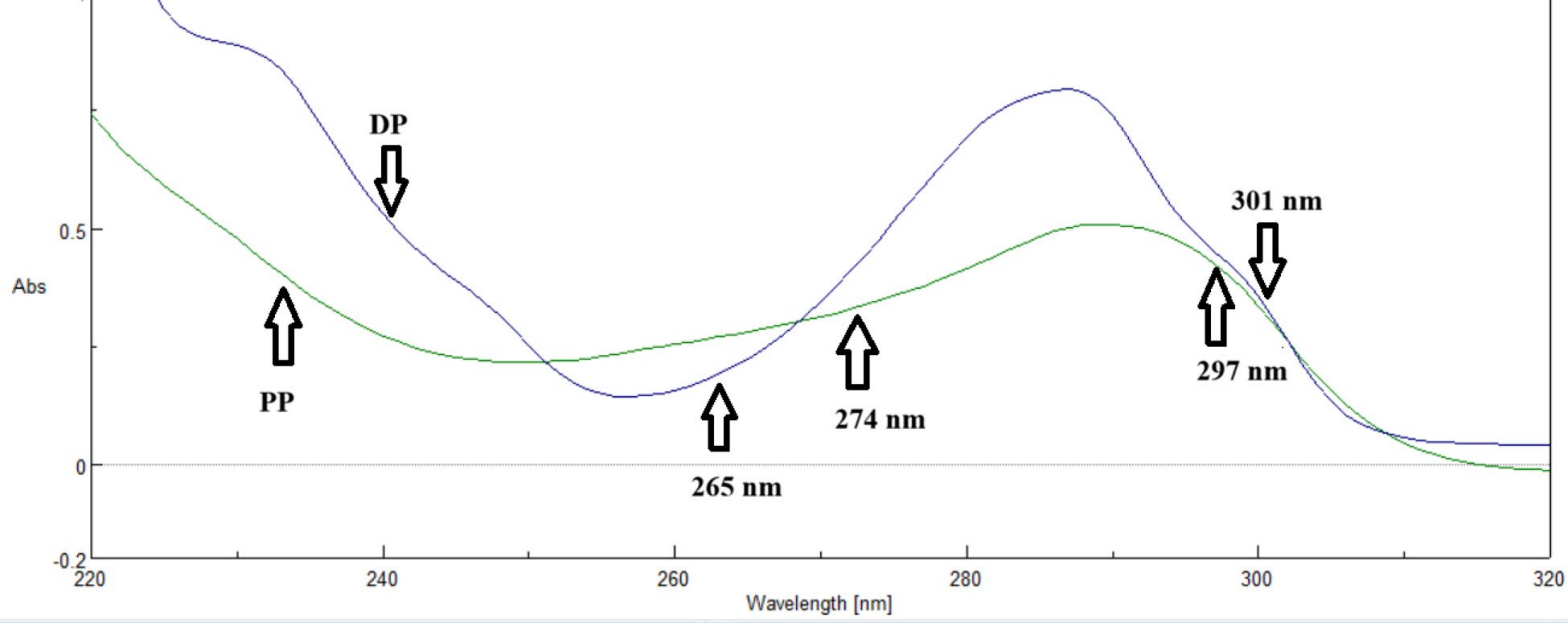
Overlain zero-order absorption spectra of DP (5.0 µg/mL) and PP (5.0 µg/mL); illustrating the determination of DP at (265.0–301.0 nm) while PP at (274.0–297.0 nm) by dual wavelength method.

(b) Dual Wavelength method (DW) for determination of DP and PP:

Dual-wavelength spectrophotometry has been developed and proven to simultaneously estimate diverse medication combinations. The dual wavelength approach is used to remove interference caused by other drugs ' absorption at a single drug’s sample wavelengths. Two wavelengths are chosen for each medicine so that the difference in absorbance is zero for the second drug^31^. The 200–400 nm range was used to scan the spectra of the generated standard solutions. The absorbance difference readings for DP (265.0 and 301.0 nm), where the absorbance difference was zero for PP, and PP (274.0 and 297.0 nm), where the absorbance difference was zero for DP, are displayed in (Fig. 3). The regression equations were computed and presented in (Table 1).

DW of DP = 0.0182 C + 0.0011 *r* = 0.9996

DW of PP = 0.0186 C + 0.0036 *r* = 0.99998

where r is the correlation coefficient and C is the drug’s concentration (µg/mL).

**Fig. 4:**
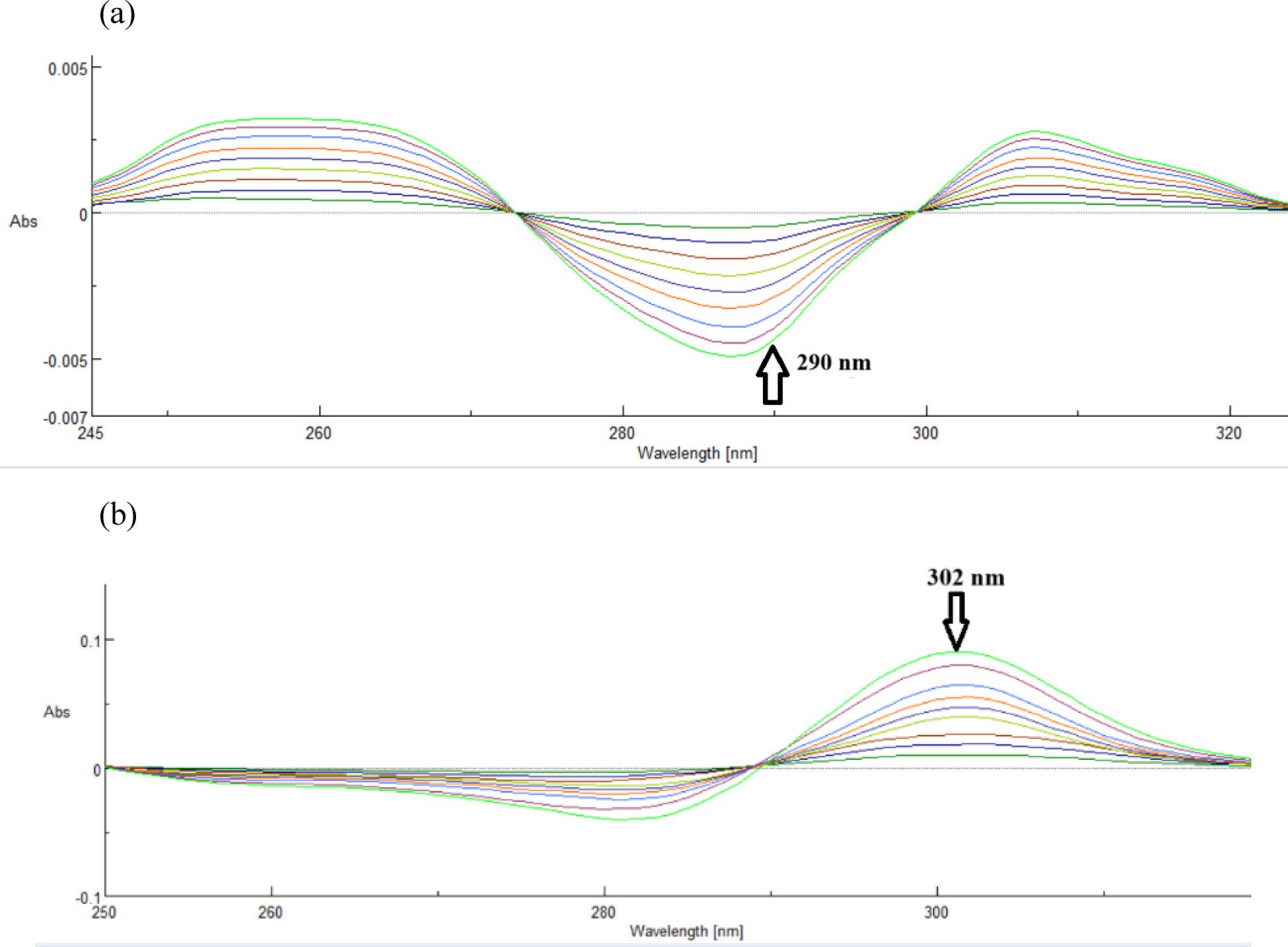
(**a**) First-order derivative of DP (1.0–9.0 µg/mL) determined at 290.0 nm, (**b**) First-order derivative of PP (1.0–9.0 µg/mL) determined at 302.0 nm.

(c) First-order derivative method for determination of DP and PP:

Because derivatization can minimize spectral background interferences and separate unresolved signals, with this method, one analyte can be quantified in the presence of others without first separating or purifying them. The instrument mode was used to convert the absorption spectra to first-order derivative spectra. Drugs with zero crossing points were chosen for additional investigation using overlapping first-order derivative spectra with a scaling factor = 9 for DP and PP. At the initial wavelength of 290.0 nm (zero crossing of PP), DP exhibited a notable absorption. PP demonstrated significant absorbance at the second wavelength, 302.0 nm (zero crossing of nm (zero crossing of DP), as seen in (Fig. 4). (Table 1) displays the regression equations that were computed.

D^1^ of DP = 0.00625 C + 0.000472 *r* = 0.9996

D^1^ of PP = 0.00882 C + 0.000588 *r* = 0.99998

where r is the correlation coefficient and C is the drug’s concentration (µg/ml).

**Fig. 5:**
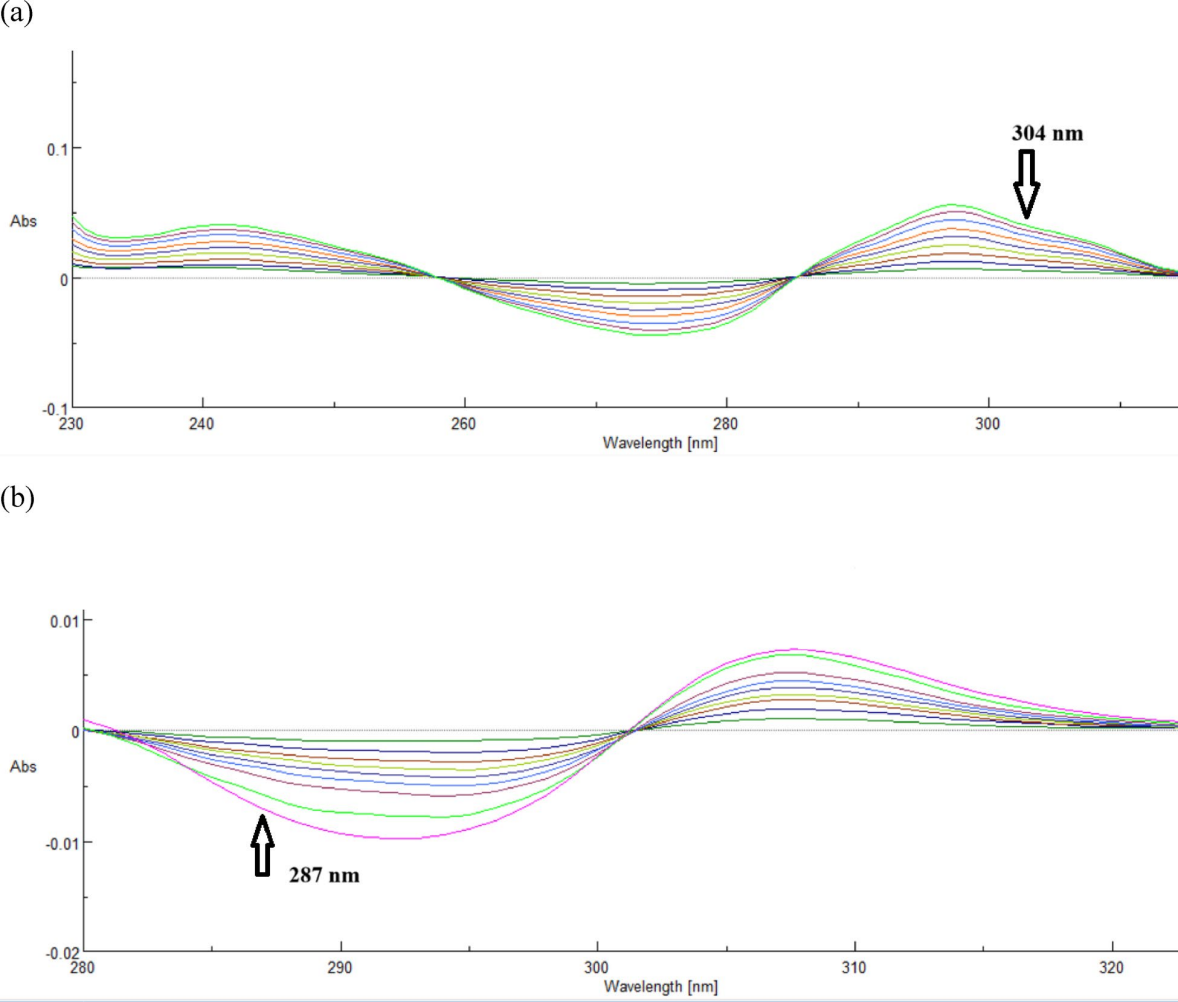
(**a**) Second-order derivative of DP (1.0–9.0 µg/mL) determined at 304.0 nm, (**b**) Second-order derivative of PP (1.0–9.0 µg/mL) determined at 287.0 nm.

(d) Second-order derivative method for determination of DP and PP:

Second derivative spectra are shown by the presence of two sharp peaks and valleys. It is possible to identify absorbance bands even in a narrow wavelength range when there are two or more overlapping peaks. The second derivative and concentration are exactly proportional. A big ratio (d^2^A/dλ^2^) indicates better sensitivity. If the background is a linear or quadratic function of wavelength, using a D^2^ or higher order derivative will remove interference and improve the recovery percentage. Second-order derivative spectra with scaling factors = 19 and 9 for DP and PP, respectively, were utilized to choose zero crossing sites of medications for future investigation. At the first wavelength (zero crossing of PP), which was 304.0 nm, DP demonstrated a notable absorption. PP demonstrated significant absorbance at the second wavelength, 287.0 nm (zero crossing of nm (zero crossing of DP), as illustrated in (Fig. 5). (Table 1) displays the regression equations that were computed.

D^2^ of DP = 0.000565 C + 5.83333 *r* = 0.9998.

D^2^ of PP = 0.000755 C − 0.000231 *r* = 0.9999.

where r is the correlation coefficient and C is the drug’s concentration (µg/ml).

### Validation of proposed techniques by ICH guidelines^20^

- Linearity: Linearity is the ability of the procedure to yield test results that are precisely proportionate to the analyte concentration in samples. This was calculated to be in the range 1.0–9.0 µg/mL for both drugs. Beer-Lambert’s law was followed by these solutions in the above concentration range with a 0.999 regression as in (Table 1).
- Accuracy: The correctness of an analytical method is determined by the degree of agreement between the value found and the value that is accepted as either a conventional true value or an acceptable reference value. This is sometimes called trueness. (Table 1) showed the calculated mean of five distinct percent recoveries and (Table S1) in details.
- Precision: The precision of an analytical method is defined as the degree of agreement between a set of measurements made by repeatedly sampling the same homogenous sample under specific conditions. Precision could be three levels of consideration: reproducibility (across labs, collaborative research, typically used to standardize approach), intermediate accuracy (differences that occur inside laboratories: different analysts, different equipment, different days), and repeatability (under the same operational circumstances for a brief period). RSDs (%) were then estimated, and the approved values were determined, as shown in (Table 1).
- LOD and LOQ: To determine the method’s sensitivity,

LOD is the smallest detectable but not usually quantifiable level of analyte in a sample. In accordance with ICH guidelines, the slope and SD of the response can be utilized to calculate the limit of detection using the following formula.

LOD = 3.3 × (SD of the response/slope)

LOQ is the lowest analyte concentration in a sample that can be identified under specified experimental conditions with a reasonable level of precision and accuracy. According to ICH recommendations, the following equation can be used to determine the limit of quantification.

LOQ = 10 × (SD of the response/slope)

SD is the intercept’s standard deviation. (Table 1) showed adequate values for the validation parameters listed above.

(e) Specificity: is the ability to conclusively assess the analyte in the presence of possibly anticipated components. Typically, these could include pollutants, degradants, and matrix. Using lab-prepared mixtures of DP and PP at different ratios and concentrations, within the linear range, we assessed specificity. (Table 2) shows that the outcomes were satisfactory.
(f) Determination of DP and PP in Their Pharmaceutical Dosage Form:

**Table 2:** Determination of DP and PP in laboratory-prepared mixtures by the developed univariate spectrophotometric methods.

%Recovery									
Conc(µg/mL)		DF		DW		D^1^		D^2^	
DP	PP	DP	PP	DP	PP	DP	PP	DP	PP
1.0	4.0	98.92	99.89	99.18	98.87	98.76	98.67	100.59	98.20
3.0	4.0	101.68	101.38	98.84	98.93	101.19	100.37	100.79	101.51
1.0	6.0	100.29	99.37	99.23	101.95	101.96	100.93	101.48	100.76
5.0	2.0	99.48	99.82	101.43	98.76	100.38	99.79	98.0	102.03
4.0	8.0	98.82	100.46	98.73	98.09	101.89	101.21	101.69	100.38
Mean ± SD		99.84 ± 1.18	100.19 ± 0.77	99.47 ± 1.11	99.61 ± 1.51	100.83 ± 1.32	100.19 ± 1.01	100.48 ± 1.54	100.58 ± 1.48

The univariate approach was used to analyze DP and PP in the Pantosec-D dose form. (Table 3) provides accurate and reliable data results for applying each procedure to the dosage form by the direct method. The standard addition approach yielded satisfactory results, exhibiting no influence from the excipients as shown in (Table 4).

**Table 3:** Results obtained by the developed methods for the determination of DP and PP in pantosec-D formulation by the direct method.

Pharmaceutical formulation			%Found ± SD ^a^			
	Drug	Conc µg/ml	DF	DW	D^1^	D^2^
Pantosec-D Tablets (each tablet contains (10.0 mg DP and 40.0 mg PP)	DP	1.0	99.04 ± 1.09	99.73 ± 1.98	101.96 ± 1.6	100.59 ± 1.77
	PP	4.0	99.09 ± 1.32	98.38 ± 1.36	98.58 ± 0.87	99.78 ± 0.68

^a^ Average of five replicates.

**Table 4:** Results obtained by the developed methods for the determination of DP and PP in the application of the standard addition technique.

Claimed, µg/mL		Pure added, µg/ml		Standard addition technique							
				%Recovery of the pure added amount ^a^							
				DF		DW		D^1^		D^2^	
DP	PP	DP	PP	DP	PP	DP	PP	DP	PP	DP	PP
1.0	4.0	2.0	1.0	98.0	99.14	98.92	98.19	99.78	99.89	98.97	100.35
		4.0	2.0	100.02	98.0	98.79	98.0	101.89	100.97	101.25	100.46
		5.0	3.0	101.43	98.46	99.87	101.20	100.71	101.32	100.83	101.38
Mean ± SD				99.74 ± 1.96	98.48 ± 0.66	99.19 ± 0.58	99.05 ± 1.88	100.79 ± 1.058	100.73 ± 0.74	100.35 ± 1.22	100.73 ± 0.57

^a^ Average of three replicates.

### Statistical comparisons

The reported spectrophotometric technique for DP and PP^12^ was statistically compared to the obtained results from DP and PP in their manufactured pharmaceutical formulation using the recommended procedures. The calculated and tabulated t and F values, as shown in (Table 5), confirmed that there are no statistical variances between them.

**Table 5:** Statistical comparison of the adopted methods and the reported method for DP and PP determination in their pharmaceutical dosage form.

	DP					PP				
Parameter	DF	DW	D^1^	D^2^	Reported method (23)^c^	DF	DW	D^1^	D^2^	Reported method (23)^c^
Mean^a^	100.49	100.48	100.36	100.19	100.87	100.06	100.36	99.95	100.42	101.09
SD	0.42	0.58	0.44	0.55	1.107	0.38	0.70	0.79	0.72	0.823
Variance	0.176	0.336	0.194	0.298	1.225	0.142	0.494	0.638	0.525	0.677
Student’s t-test (2.31)^b^	0.72	0.70	1.75	2.03		2.29	1.52	2.22	1.37	
F-test (6.39)^b^	6.25	3.64	3.50	2.27		4.77	1.37	1.06	1.29	

^a^ Average of five experiments (*n* = 5) for the adopted methods and three experiments (*n* =3) for the reported method.

^b^ The values between parentheses are the theoretical values for t and F at *P* = 0.05.

^c^ The reported spectrophotometric technique simultaneous equation and Q- analysis UV based on measurement absorptivity at 216, 287 and 290 nm, respectively. Linearity lies between (1–15) µg/mL for domperidone and (0–50) µg/mL for pantoprazole.

### Assessing the environmental impact of different analytical procedures

It is crucial to evaluate the environmental impact of different analytical techniques based on their adherence to green chemistry principles, rather than depending on the authors’ to evaluate the effects on the environment of various analytical processes based on their adherence to green chemistry principles, rather than relying on the authors’ subjective perspectives or assumptions. To assess the developed methodologies’ environmental friendliness, we used Eco-scale, MoGAPI, AGSA, CaFRI, BAGI, CACI and WAC metrics.

## Analytical eco-scale system (penalty point)

Each item on the scale is given a penalty point in this semi-quantitative assessment method; instrumentation, energy usage, solvents utilized, and waste all receive penalty points for their potential impact on the analytical process^32^. To find the method’s base value, subtract the sum of produced and predictable hazards from 100. When these standards are applied to the created procedures, an eco-score of 90 was developed, as shown in (Table 6a), indicating that it is an excellent green method. We compared with another HPLC method^10^ as shown in (Table 6b), but our developed method is better.

**Table 6:** The eco-scale tool is used to evaluate the greenness of the developed methods.

Parameters	Penalty points
(a)	
Ethanol	12
Instrument	Penalty points
Energy (UV/V spectrophotometer) (≤ 0.1 kWh per sample)	0
Occupational hazards (analytical process hermetization)	0
Waste (1–10 ml, no treatment)	5
Analytical eco-scale total score^a, b^	87
Comment	Excellent green analysis
(b)	
Methanol	6
Acetonitrile	8
Phosphoric acid	2
Hydrogen peroxide	4
Energy consumption	1
Occupational hazards (analytical process hermetization)	1
Waste treatment	3
Waste treatment	3
Analytical eco-scale total score^a, b^	72
Comment	Acceptable green analysis

^a^ Analytical Eco-Scale total score = 100 - total penalty points.

^b^ If the score is > 75, it represents excellent green analysis. If the score is > 50, it represents acceptable green analysis. If the score is < 50. It represents inadequate green analysis.

## Green assessment of the developed methods

### Modified green analytical procedure index (MoGAPI)

**Fig. 6:**
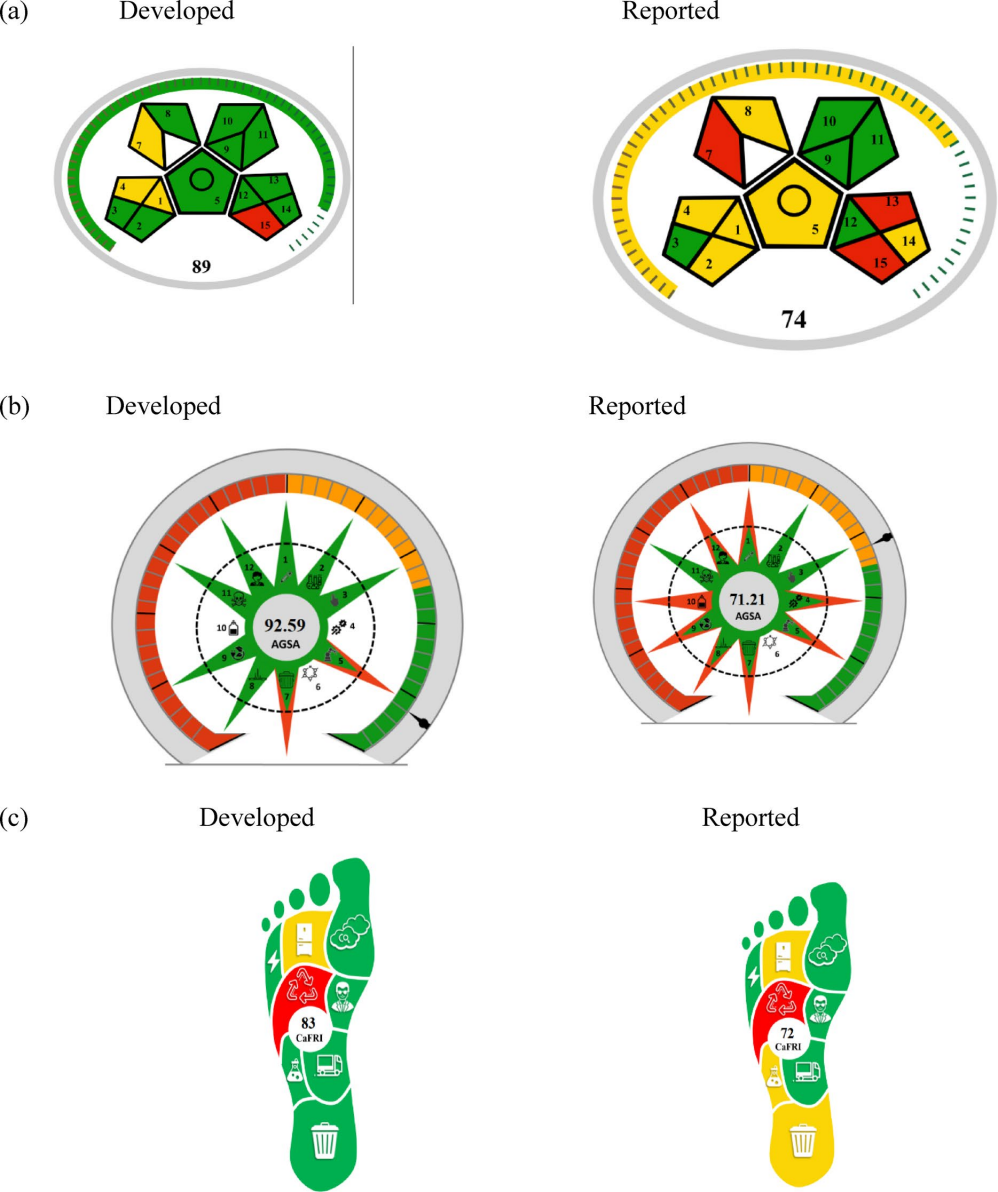
Green assessment of the developed method by MoGAPI (**a**), AGSA (**b**), and CaFRI (**c**) pictograms.

Although MoGAPI is comparable to the Green Analytic Procedure Index (GAPI), the latter does not provide a total score that enables technique comparison, making MoGAPI superior. A modified GAPI tool (MoGAPI) and software have been developed and used in this study to address the drawbacks of the current GAPI measure. The software makes it easier and faster to use, but the instrument that is being provided offers a more accurate assessment of greenness^33^. The MoGAPI evaluation was carried out using MoGAPI, which has 15 parameters represented by five pentacle forms and is accessible at bit.ly/MoGAPI. It is intended to measure and assess the environmental effects of every phase of an analytical procedure, including waste management, instruments, solvent and reagent health risks, and sample preparation. The system is color-coded, with red denoting high environmental damage, yellow denoting medium impact, and green denoting moderate impact. Green zones were found in energy and equipment type (UV spectrophotometry detection), but red zones were found in solvent use (methanol, TEA). As seen in (Fig. 6a), the majority of sectors displayed green status, and the MoGAPI pictogram in the developed approach included a value score of 89. We compared with another HPLC method^10^, but our developed method is better as shown in (Fig. 6a).

### The analytical green star area (AGSA)

Similar to the Analytical GREEnness metric, it does not classify analytical procedures based on overall scores, lacks an analogous statistic in the Green Chemistry (GC), and is less resistant to human bias. To close these gaps, the Analytical Green Star Area (AGSA) offers a comprehensive, integrated grading system and an easily comprehensible evaluation procedure that determines whether the suggested techniques align with the environmental green concept. Respecting the 12 Principles of the GAC. Furthermore, AGSA is an extension of a similar GC^34^ statistic. According to this page, the suggested AGREE pictograms are shown (http://bit.ly/AGSA2025). A free software program called AGSA is used to create a circular pictogram with twelve parts, each of which represents one of the twelve GAC features. Depending on how the analysis approach impacts the environment, each segment of the pictogram has a different color, ranging from deep green (indicating the lowest ecological impact) to deep red (indicating the highest ecological impact). AGSA’s pictogram (92.59) includes a valuation score in the center, and we compared it with another HPLC method^10^, but our developed method is better as shown in (Fig. 6b).

### Carbon footprint

Growing levels of greenhouse gases in the atmosphere are disrupting the environment and causing serious global warming and its aftereffects. Following the rule that only data that can be measured can be managed, the carbon footprints of various bodies, companies, and activities worldwide reveal their greenhouse gas intensity. To present a more comprehensive perspective on sustainability, the analytically created method’s carbon footprint was evaluated using bit.ly/CaFRI, yielding a score of 83, and we compared our method with another HPLC method^10^, but our developed method is better, as illustrated in (Fig. 6c). Our spectrophotometric approach proved practical and applicable.

## Blueness assessment of the developed methods

### The blue applicability grade index (BAGI)

**Fig. 7:**
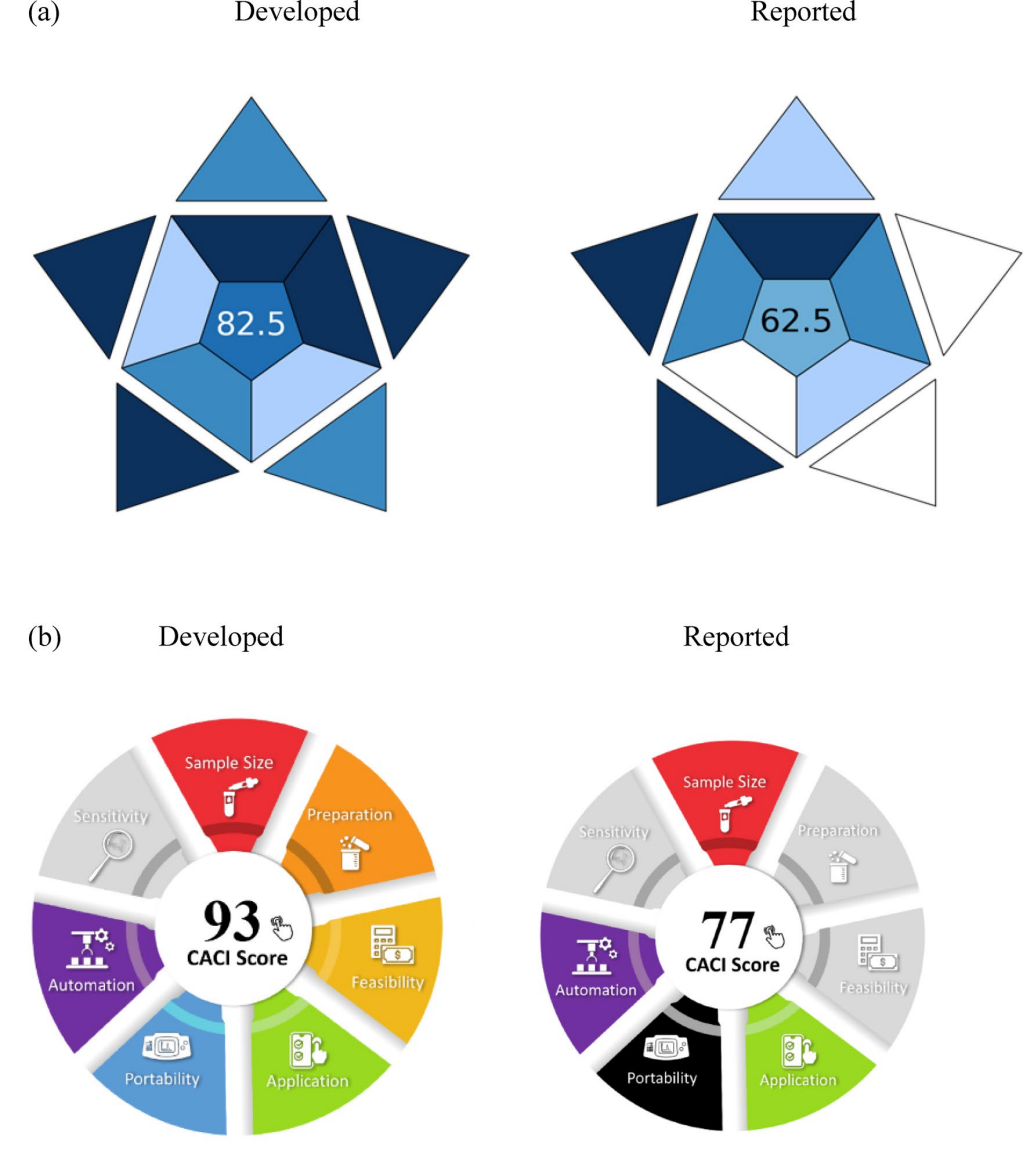
Blueness assessment of the developed method by BAGI (**a**) and CACI (**b**) pictograms.

The Blue Applicability Grade Index (BAGI) ranks analytical chemistry procedures on a scale of 25 to 100 based on their practicality. A higher grade suggests a more realistic strategy. It permits quick evaluation of the strengths and limitations of a method in terms of its applicability, as well as comparing the performance of other analytical methods^35^. The strategy must score higher than 60 to provide a green method. BAGI’s validation criteria include the nature of the analysis, equipment, various substances, sampling demands, sample size, sample handling capacity, number of samples processed per hour, automation level, preconcentration needs, and required reagents/materials. Each category is assigned a color based on these norms, ranging from dark blue (indicating great compliance, application, or suitability) to white (indicating non-compliance). With a BAGI score of 82.5, our spectrophotometric technique exhibited usefulness and applicability, and we compared our method with another HPLC method^10^, but our developed method is better as shown in (Fig. 7a).

### Click analytical chemistry index (CACI)

The CACI is built on providing a practical, efficient, and understandable framework for evaluating and contrasting analytical procedures, and it was inspired by the simplicity and reliability of click chemistry. Important characteristics that are graded to highlight the method’s compliance with these guidelines are sample size, preparation, practicality, application, portability, sensitivity, and automation. Method validation is an essential precondition before CACI may evaluate the methodology. The method’s performance in each area is shown by the color code of the CACI pictogram. This framework is easy to use and allows users to quickly assess the method’s benefits and drawbacks across multiple factors^36^. As seen in (Fig. 7b), a colored pictogram score of 93 denotes excellent performance, gray denotes intermediate performance, and black denotes subpar performance or inability to meet the desired requirements. In addition to comparing it with another HPLC method^10^, which shows that our developed method is better.

## The multi-color assessment (MA) tool of the developed methods

**Fig. 8:**
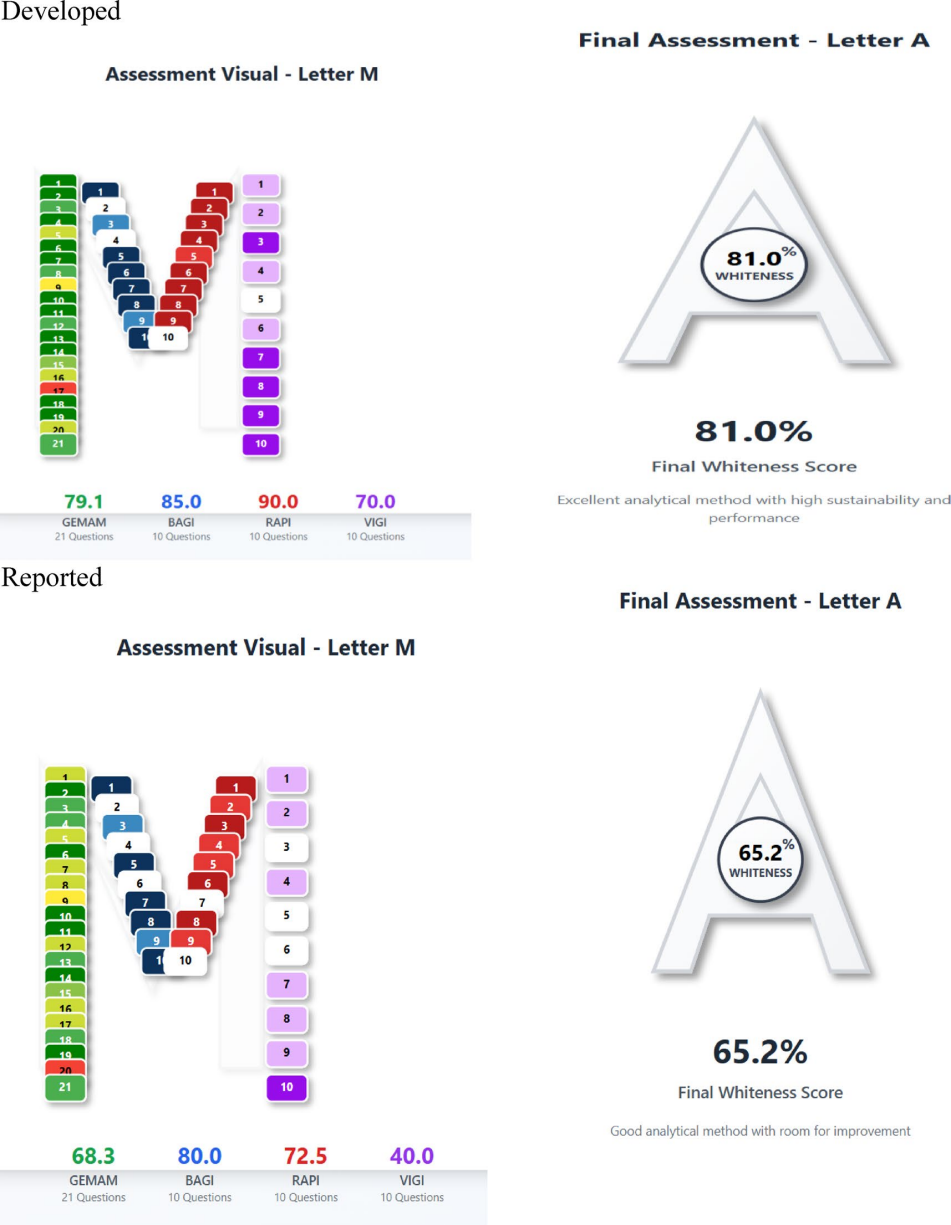
White assessment of the suggested and reported method by MA Tool pictograms.

The Multi-Color Assessment (MA) Tool is introduced in this study as a comprehensive web-based platform that consolidates four established assessment frameworks into one evaluation system. The MA Tool combines the Green Evaluation Metric for Analytical Methods (GEMAM), Blueness Assessment Graphical Index (BAGI), Redness Analytical Performance Index (RAPI), and Violet Innovation Grade Index (VIGI) via a structured assessment protocol consisting of 51 questions. Users fill out domain-specific questionnaires assessing environmental impact, operational feasibility, analytical performance, and technological innovation. For each dimension, the platform produces individual scores and computes a composite “Whiteness Score” that reflects the overall sustainability of the method. Moreover, this work presents a novel scoring direction grounded in the principles of Analytical Quality by Design (AQbD). This represents a crucial advancement not covered by prior tools, thereby enhancing the scientific and regulatory significance of the platform. This addition demonstrates an advancement beyond environmental and practical scoring, incorporating quality assurance principles into the sustainability assessment for the first time. An interactive 3D color-segmented typographic display visualizes the results, with each dimension represented by different colored segments. By pinpointing particular strengths and weaknesses, the tool facilitated method enhancement and informed choices. The MA Tool offers real-time scoring and automated PDF report generation, with no need for installation. This platform, which can be accessed freely at https://effervescent-naiad-a47bbd.netlify.app, promotes the principles of White Analytical Chemistry (WAC) by offering the first cohesive framework for thorough evaluation of analytical methods, aiding method developers and regulatory decision-making processes. As far as we know, the MA Tool is the first fully integrated digital platform that is freely available and combines aspects of greenness, practicality, performance, and innovation into a real-time sustainability scoring system for analytical methods^37^. In addition to comparing it with another HPLC method^10^, which shows that our developed method is better as seen in (Fig. 8).

## Conclusion

The described methods for determining DP and PP in binary mixes and combined dose forms are environmentally friendly, accurate, and easy. They do not require any sophisticated sample pretreatment. A statistical comparison of the applied methodologies and the reported HPLC procedures revealed that there is no significant variation in precision and accuracy. Several greenness assessment indicators were used to evaluate how environmentally friendly they are. The implemented strategies included the Analytical eco-scale system, MoGAPI, AGSA, CaFRI, BAGI, CACI and MA approaches. The use of a quantitative analytical green approach helps to protect from dangerous environmental exposure and analyzers. Accordingly, these methods are suitable for use in quality control laboratories without liquid chromatographic apparatus.

## Supplementary Information

Below is the link to the electronic supplementary material.

Supplementary Material 1

## Data Availability

Data available on request All data supporting the findings of this study are available within the paper and its Supplementary Information. For further information regarding the data used in this study, Please contact: [Mona Abd Elnasser LabibElsayed], [[monaabdelnasser89@gmail.com](mailto: monaabdelnasser89@gmail.com)].
