# Supplementary material for: Unveiling advanced green assessment of simple and cost effective spectrophotometric determination of domperidone and pantoprazole for gastrointestinal disorders
Source: Sci Rep. 2026 Jan 26;16:3475. doi: 10.1038/s41598-026-35089-1 (PMC12848105; doi:10.1038/s41598-026-35089-1)
Supplement: Supplementary file 1 — Supplementary Material 1 [file 41598_2026_35089_MOESM1_ESM.docx]

**Table S1**. Accuracy of DP (a) and PP (b) of the zero order:

(a)

| concentrations taken(μg/mL) | concentrations found | corresponding % recovery | (Mean ± SD) |
| --- | --- | --- | --- |
| 1 | 0.993 | 99.26 | 100.63± 0.44 |
|  | 1.001 | 100.15 |  |
|  | 1.010 | 101.04 |  |
| 4 | 4.055 | 101.39 |  |
|  | 4.082 | 102.06 |  |
|  | 4.019 | 100.49 |  |
| 5 | 5.045 | 100.92 |  |
|  | 5.091 | 101.83 |  |
|  | 4.975 | 99.51 |  |
| 6 | 6.064 | 101.08 |  |
|  | 6.046 | 100.78 |  |
|  | 5.949 | 99.14 |  |
| 8 | 8.154 | 101.93 |  |
|  | 7.957 | 99.47 |  |
|  | 8.038 | 100.48 |  |

(b)

| concentrations taken(μg/mL) | concentrations found | corresponding % recovery | (Mean ± SD) |
| --- | --- | --- | --- |
| 2 | 2.033 | 101.67 | 99.49 ± 0.69 |
|  | 1.968 | 98.42 |  |
|  | 1.997 | 99.86 |  |
| 3 | 3.041 | 101.39 |  |
|  | 3.006 | 100.19 |  |
|  | 2.934 | 97.8 |  |
| 4 | 3.978 | 99.46 |  |
|  | 4.065 | 101.62 |  |
|  | 4.072 | 101.81 |  |
| 5 | 4.843 | 97.86 |  |
|  | 4.915 | 98.30 |  |
|  | 4.987 | 99.74 |  |
| 6 | 5.923 | 98.72 |  |
|  | 6.068 | 101.13 |  |
|  | 5.995 | 99.93 |  |
